# High energy clavicle shaft fractures consistently occur at the inflection point: defining morphology and correlation with fracture patterns

**DOI:** 10.1016/j.jseint.2026.101623

**Published:** 2026-02-05

**Authors:** Rhiana Rivas, Elizabeth Muhammad, Ana Love, Ann Laurie Wells, Julie Mekhail, Jorge Orbay, Deana Mercer

**Affiliations:** a Department of Orthopedic Surgery, University of New Mexico School of Medicine, Albuquerque, NM, USA; b Burrell College of Osteopathic Medicine, Las Cruces, NM, USA; c Miami Bone and Joint Institute, Miami, FL, USA

**Keywords:** Clavicle fracture, Inflection point, Clavicle morphology, 3D reconstruction, Orthopedic trauma, Morphometric analysis, CT imaging, Midshaft clavicle

## Abstract

**Background:**

Midshaft clavicle fractures are the most common subtype of clavicle injuries, yet the morphologic basis for their consistent location remains unclear. This study aims to define the anatomical inflection point of the clavicle and investigate its correlation with fracture location, hypothesizing that most high-energy midshaft fractures occur at this morphologically consistent point of curvature transition.

**Methods:**

A retrospective review identified 115 patients with high-energy clavicle fractures confirmed on computed tomography imaging from 2011 to 2023. After applying exclusion criteria, 100 fractures were reconstructed into three-dimensional models. Morphometric analysis was performed using image-modeling software. Polynomial equations were fit to the clavicle centerlines to calculate inflection points using second-derivative analysis. Morphologic variables, including arc lengths, radii of curvature, and fracture distance from the medial border, were measured. Statistical comparisons were made by fracture type (Allman classification), gender, and morphometric parameters using analysis of variance, *t*-tests, and nonparametric tests.

**Results:**

Of the 115 analyzed clavicles, 78% were midshaft fractures. Fractures consistently occurred at approximately 60% of the clavicle's total length from the medial border, corresponding to the inflection point of curvature. Medial arcs averaged longer lengths and larger radii of curvature than lateral arcs. Gender analysis revealed shorter clavicle lengths and differing arc ratios in female patients (*P* < .05). No significant difference in fracture location was observed by gender or laterality.

**Conclusion:**

High-energy clavicle fractures consistently occur at the anatomical inflection point, a morphometrically distinct region where curvature transitions from medial to lateral. Recognition of this consistent fracture location may improve surgical planning, implant design, and biomechanical modeling.

Clavicle fractures are among the most common orthopedic injuries, accounting for 2.5%-10% of all fractures.^4^^,^^6^^,^^13^^,^^15^ They result from acute trauma to the shoulder, such as direct falls or high-energy impacts.^11^ Fracture locations can be defined by the Allman classification system, which divides the clavicle into thirds and defines group I fractures as the middle third, group II fractures as the lateral or distal third, and group III as the proximal or medial third.

Management of displaced middle-third clavicle fractures, such as those resulting from high-energy trauma, presents a significant challenge to the orthopedic surgeon. The literature supports that operative management with either plating or intramedullary pinning results in lower nonunion rates when directly compared to nonoperative management within the same cohorts.^17^ Though it is well documented that the middle third of the clavicle is where fractures tend to occur the most (accounting for 70%-80% of all clavicle fractures^2^), there is a shortage of literature that quantifies clavicular fractures with respect to clavicular morphology. One such study is by Agyeman et al^1^ who looked closely at midshaft fractures in 166 patients post-operative radiographs, reporting 90% of midshaft fractures were centered lateral to the midpoint and 64% were completely lateral to the midpoint. The authors further defined a “fracture zone” that was localized 42% of the distance from the lateral border, or 58% percent from the medial border. Building on this foundational work, our study aims to analyze computed tomography (CT) scans of clavicle shaft fractures to further clarify trends in fracture patterns and to discern correlations between fracture location and clavicle morphology. We hypothesized that fractures occur at specific points along the clavicular body that are within an acceptable percentage range from the results of Agyeman's study. We further hypothesize that high-energy midshaft fracture patterns can be explained morphometrically and correlate to a specific anatomical region known as the inflection point and defined as the transition between the lateral and medial arc of the clavicle. This detailed investigation of clavicle fracture morphology may better inform surgical planning and plate design in the management of high-energy midshaft clavicular fractures.

## Methods

### Patient identification

**Figure 1:**
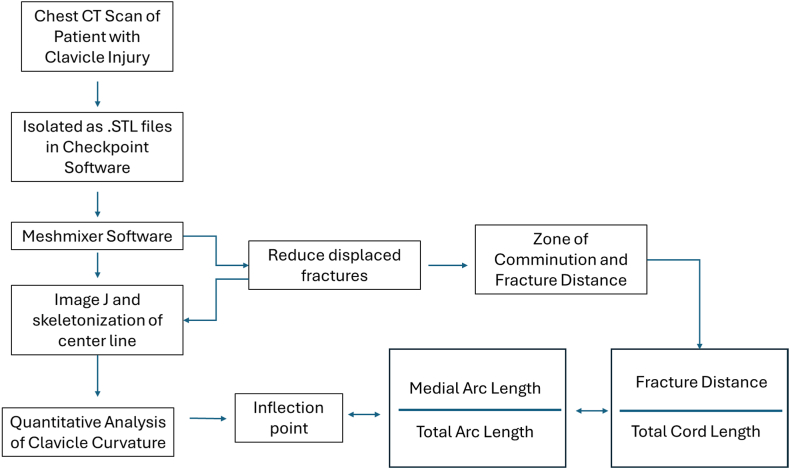
Methodology flowchart demonstrating methodology of specimen analysis. *CT*, computed tomography.

We identified 115 patients who presented to the emergency department from 2011 to 2023 with confirmed clavicle fractures on CT using our institution's electronic medical record system using Current Procedural Terminology codes 23500, 23515, 23480, and 23515 with 20680; International Classification of Disease, Tenth Revision codes: S42.∗; and International Classification of Disease, Ninth Revision codes: 810.∗ and 79. CT scans were required to create three-dimensional (3D) models for assessment, with which plain film was not compatible with our modeling software; thus, these scans tended to correlate with high-energy trauma. One hundred and fifteen patients were selected from a pool of randomized patient records from the initial data query of chest CT scans (Fig. 1). Pregnant women, prisoners, and patients under 18 years of age were excluded from this study. Patients who had a confirmed clavicle fracture and corresponding chest CT scan were included. A retrospective chart review was conducted to identify the location of the clavicle fracture, injured clavicle side (left or right), fracture zone, mechanism of injury (MOI), age at time of injury, and gender.

### Image processing and modeling

**Figure 2:**
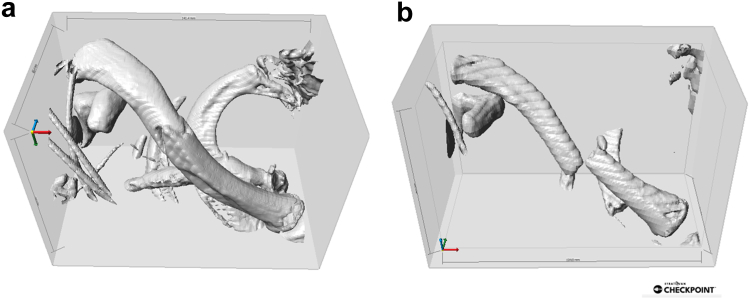
(**a**) and (**b**) show cropped and isolated clavicles from 2 different patients in checkpoint modeling software.

The corresponding patient Chest CT scans were deidentified and downloaded as Digital Imaging and Communications in Medicine files. Chest CT scans enabled the most complete capture of the clavicles. The image files were then rendered in Checkpoint software (2023; Stratovan, Sacramento, CA, USA) geometric morphometrics software as 3D models. The 3D models were cropped so that only the fractured clavicle was rendered (Fig. 2).

**Figure 3:**
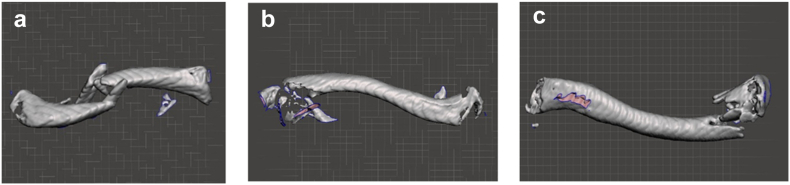
(**a**) Demonstrates a nonreduced midshaft fracture of a left clavicle, (**b**) demonstrates a lateral fracture of a left clavicle, and (**c**) demonstrates a medial fracture of a left clavicle in Meshmixer 3D software. *3D*, three-dimensional.

**Figure 4:**
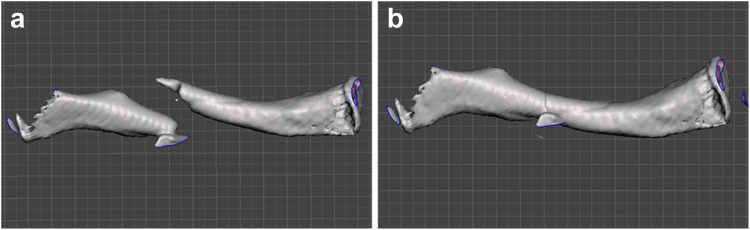
(**a**) Demonstrates a non-reduced midshaft fracture of a left clavicle in Meshmixer 3D software. (**b**) Shows the same clavicle after the fracture is reduced in the Meshmixer software. *3D*, three-dimensional.

The 3D rendering was then saved as a .STL file and imported to Meshmixer software (2025; Autodesk, San Francisco, CA, USA) for further refinement, positioning, and scale setting (Fig. 3). For consistency, all clavicles were aligned such that the lateral border of the clavicle was on the left and the medial border was on the right. If the clavicle fracture was significantly displaced, the clavicle mesh was aligned to digitally reduce the fracture near anatomically (Fig. 4). The clavicle mesh was aligned in the anterior–posterior view and positioned directly above the baseline measurement grid. The units of the grid were set to 10 mm per square. This was then saved as a two-dimensional image. The image was then imported into FIJI ImageJ software package (Version 2.16.0; FIJI, National Institutes of Health, Bethesda, MD, USA), and the fracture distance from the medial border was calculated with respect to the total length. Each sample's pixel per mm ratio was recorded at this step.

### Skeletonization and center line approximation

**Figure 5:**
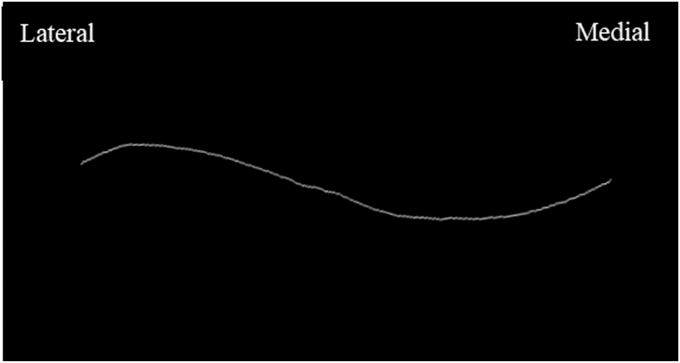
Sample of clavicular center line approximated in *ImageJ*.

The center line of the clavicle was then approximated through skeletonization using the methods described by E. Muhammad et al in a concurrent study and publication (E. Muhammad, R Rivas, A Love, A L Wells, Orbay, and Mercer, A Novel Method of Analyzing Clavicle Curvature Using Open Access Software. *Submitted, Under Review*; November 2025). Skeletonization is an image processing technique that allows for the extraction of an object's medial axis. The center line is defined as the medial axis of an object and is visually represented by the longitudinal curve of the clavicle in the coronal plane in our study. The skeletonization process was utilized to distill an object into a binary projection that preserves its shape, which has been well described for the morphometric analysis of anatomical structures.^12^ An example center line approximation of a clavicle is shown in Figure 5.

**Figure 6:**
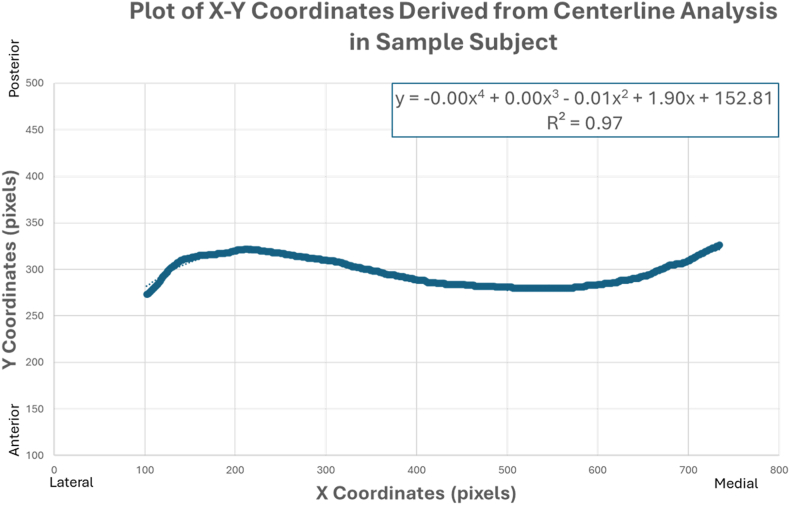
Plot of a sample subject's X-Y coordinates that were exported from the centerline analysis in ImageJ. The fourth-degree polynomial equation for this curve is represented in the upper right corner. The R-squared value validates a correlation between the curves of a clavicular center line and fourth-degree polynomial curve.

The X-Y coordinates of the center line were exported from ImageJ into Microsoft Excel (Version 2021; Microsoft Corporation, Redmond, WA, USA) to calculate the best-fit fourth degree polynomial equation. One hundred out of 115 clavicles were used to approximate the fourth degree polynomial function from the plotted coordinates (Fig. 6). The remaining 15 clavicle were severely comminuted, not accurately reconstructible, and therefore omitted.

### Variable calculation and analysis

**Figure 7:**
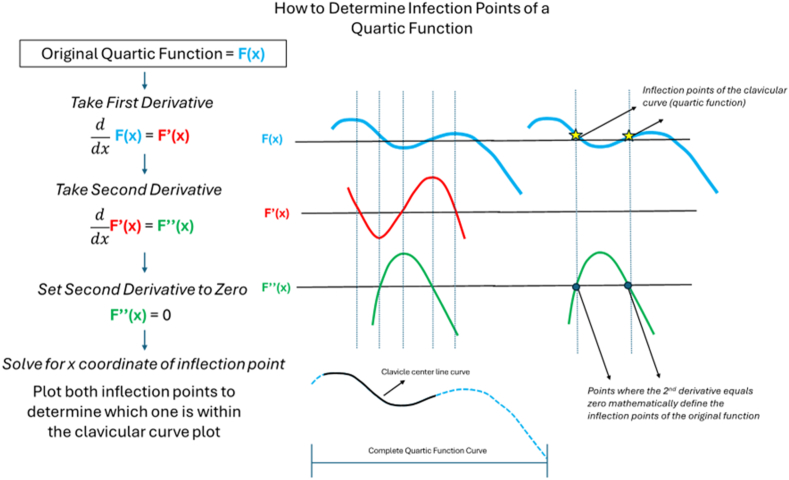
Visually demonstrates the sequence of mathematical steps taken to derive the inflection points of the clavicular curve, which is represented mathematically by a quartic function.

A fourth degree polynomial equation was determined for each clavicle curvature, and the second derivative of the equation was calculated. To determine the inflection points of a quartic function, the second derivative of the equation is set equal to zero and solved mathematically using Mathematica analytical software (2024; Wolfram, Champaign, IL, USA). Figure 7 demonstrates sample calculations and determination of the correct inflection point for the corresponding clavicular curve. Once the inflection point was determined, other values could then be derived, including medial arc length, medial chord length, medial radius of curvature, lateral arc length, lateral chord length, lateral radius of curvature, medial angle, and lateral angle using Mathematica analytical software.

**Figure 8:**
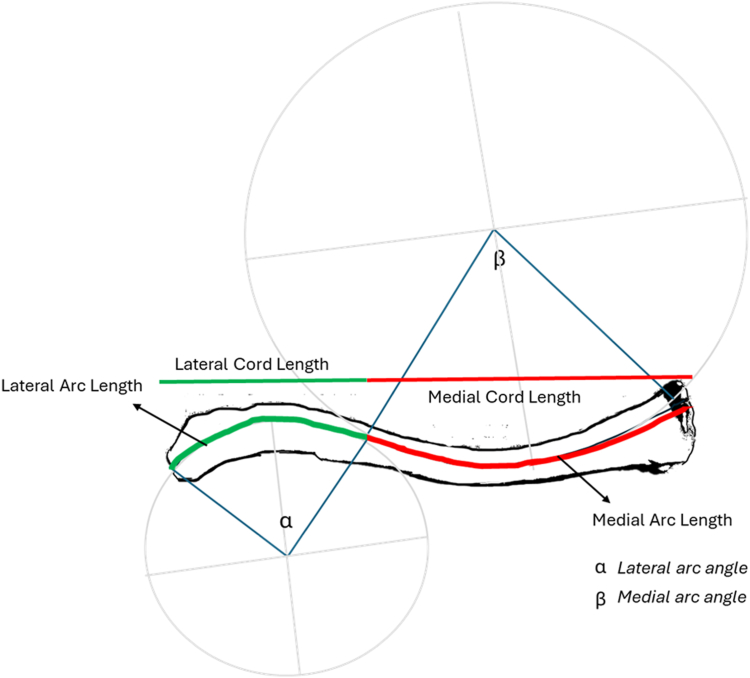
Visual diagram of the variables of interest annotated on a sample left clavicle, including lateral and medial arc length, lateral and medial chord length, lateral arc angle (alpha), and medial arc angle (beta). The figure is for visual representation only and is not derived from mathematically calculated dimensions.

The total arc length was calculated by adding the medial arc length to the lateral arc length. The total chord length was calculated by adding the medial chord length to the lateral chord length. The values of the variables were then converted to millimeters by dividing the pixel value outputs by each sample's pixels per mm ratio that was previously recorded. Figure 8 visually demonstrates the relationship between the calculated variables and the anatomic clavicle in the anterior–posterior view.

### Assessing fracture zone of comminution and fracture location

**Figure 9:**
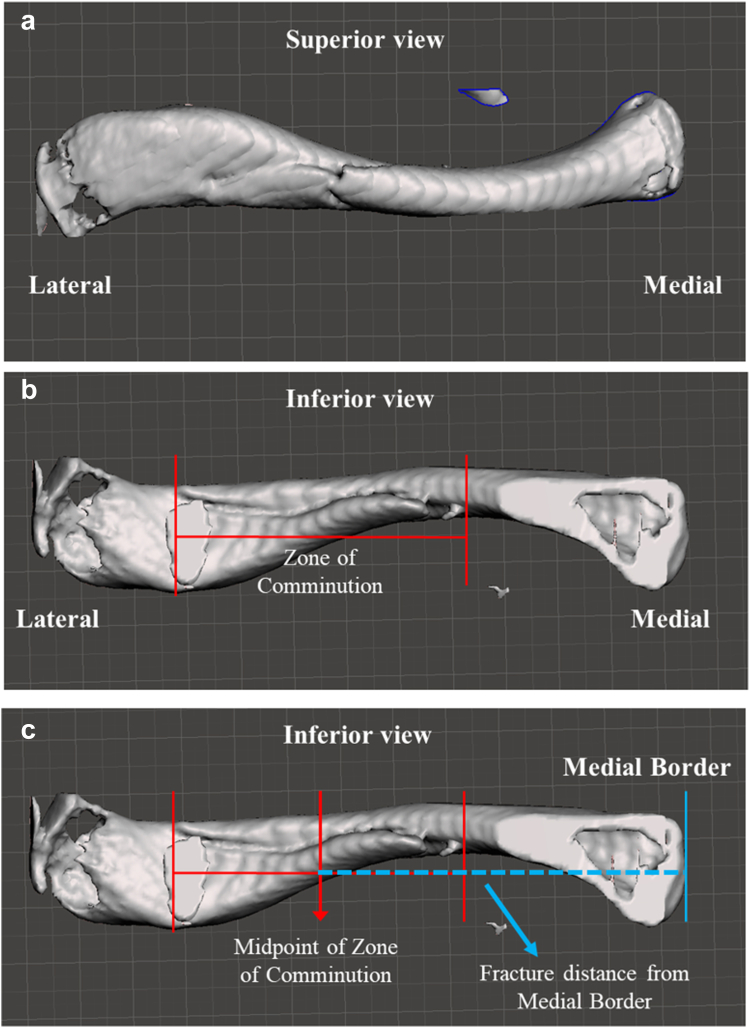
(**a**) Shows the superior surface of a right clavicle with a midshaft fracture. (**b**) Shows the inferior surface of the same clavicle, revealing a larger fracture length than what is visible from the superior surface. (**c**) Shows the zone of comminution and how the fracture length was determined relative to the medial border.

The zone of comminution was determined in the Meshmixer software package using the segment analysis tool. We spatially manipulated the 3D clavicle models to determine the distance between the lateral-most fracture segment and the medial-most fracture segment (Fig. 9, *a* and *b*). We defined the length between the lateral-most and the medial-most fracture segments as the zone of comminution. The location of the fracture was determined by measuring the distance between the midpoint of the zone of comminution and the medial border of the clavicle (Fig. 9, *c*). The ratio between this measured distance and the total chord length of the clavicle was taken and recorded. All 115 clavicle samples were measured. The measurement error is estimated to be about 0.3 mm.

### Statistical analysis

Statistical analysis was performed to compare the findings of female vs. male specimens with respect to variations in total arc length, total chord length, medial arc length to total arc length ratio, and medial chord length to total chord length ratio using Welch t-tests and Wilcoxon rank-sum tests. Statistical analysis was performed to compare the variations of the variables above between Allman group I, group II, and group III findings as well using an analysis of variance test to determine if a difference existed between the groups initially, with the statistical significance of the tests confirm with Wilcoxon signed-rank statistical tests. All statistical analysis was calculated in R Statistical Package (R Core Team v4.1.2, 2021; R Foundation for Statistical Computing, Vienna, Austria).

### Demographic analysis

The demographic information collected included sex, age, MOI, and type of clavicular fracture based on the Allman classification system. Statistical analysis was performed with chi-square test calculated in R Statistical Package.

## Results

### Qualitative variable analysis

One hundred fifteen clavicles in 115 patients were analyzed by gender, age, fracture location, displaced fracture vs. nondisplaced fracture, laterality of injury, MOI, and if the fracture required open reduction and internal fixation. The average age of the patient was 45.07 years of age. The fractures were categorized by location (lateral, midshaft, distal) and type based on the Allman (Table I).

**Table I:** Summary of variable analysis by count and percentage of cohort in 115 clavicles.

All patients	All (clavicles)	Percentage of total
Total	115	-
Female	39	33.91%
Male	76	66.09%
Displaced	112	97.39%
Left clavicle	51	44.35%
Right clavicle	64	55.65%
Midshaft fracture	90	78.26%
Lateral (distal) fracture	18	15.65%
Medial (proximal) fracture	7	6.09%

### Fracture location by gender

**Figure 10:**
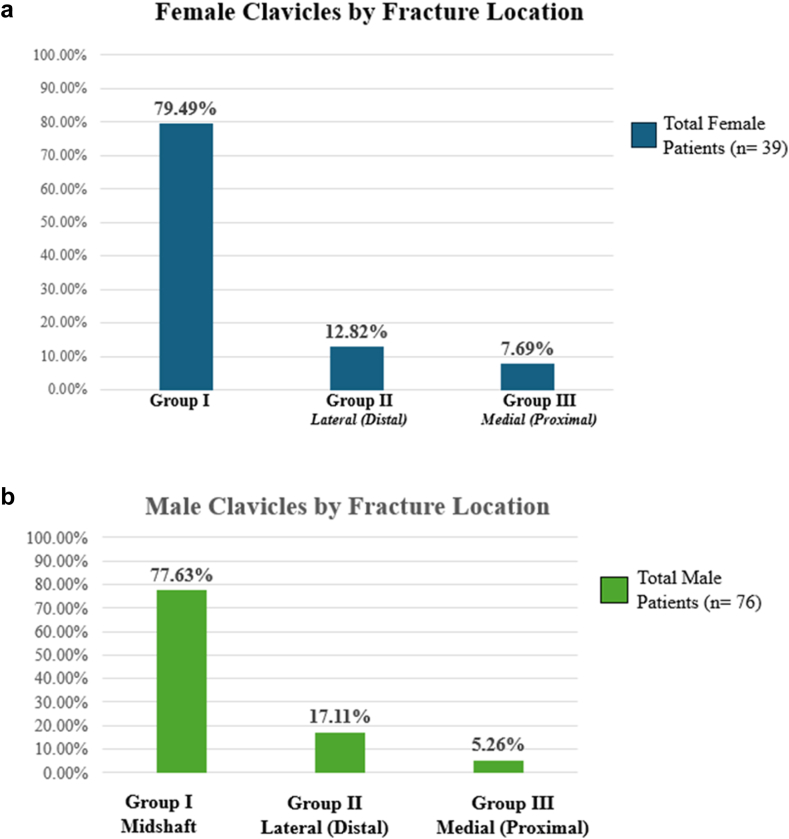
(**a**) Fracture location distribution by female patient cohort. (**b**) Fracture location distribution by male patient cohort. Results are broken down by Allman classification of fracture location.

There was no statistically significant difference in fracture location between males and females (*P* = .74). The frequency of location of clavicle fracture by gender is demonstrated in Figure 10.

### Fracture by mechanism of injury

**Figure 11:**
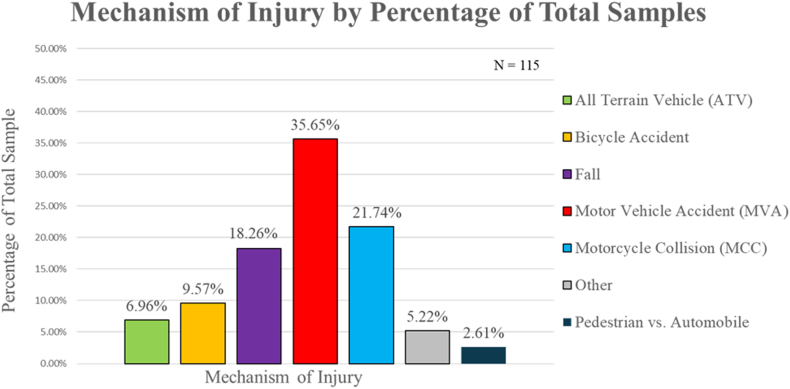
Percentage of clavicles distributed by mechanism of injury.

**Figure 12:**
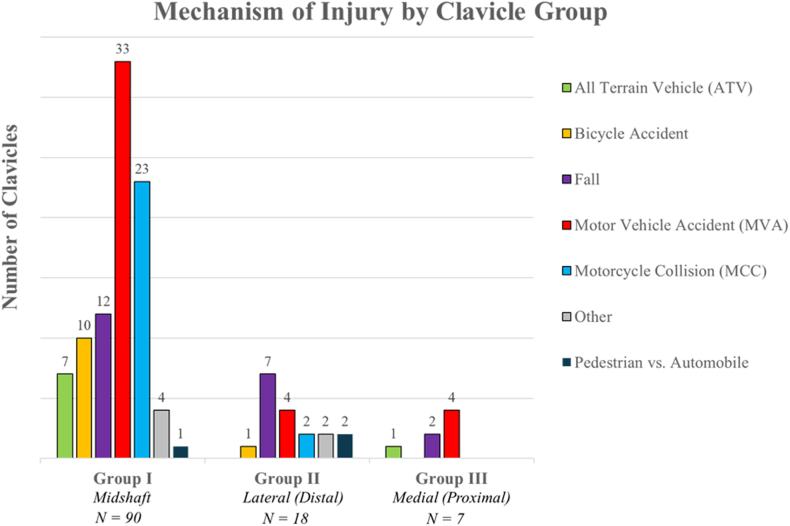
Mechanism of injury by midshaft, lateral, and medial fracture location.

The most common MOI was motor vehicle accident (MVA), followed by motorcycle collision. Bicycle accidents were the third most common MOI, followed by all-terrain vehicle accidents (Fig. 11). MVAs were the most common cause among midshaft and medial fractures, while falls were the most common MOI in lateral shaft fractures (Fig. 12). The “fall” MOI included falls from heights of ten feet or greater, falls from standing height, and falls from horse.

### Clavicle fracture location by age

**Figure 13:**
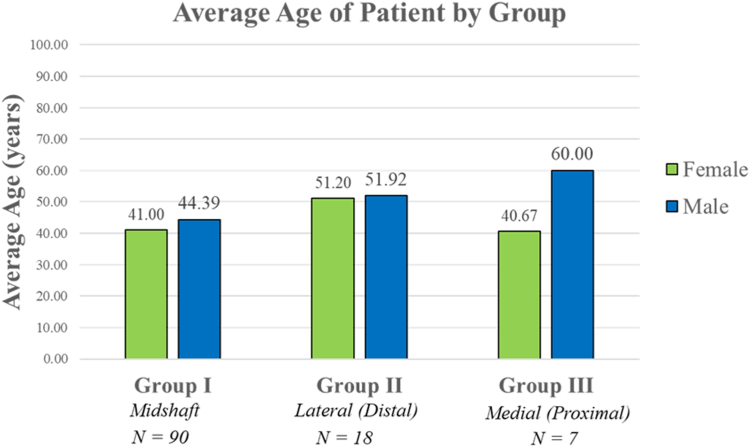
Distribution of average age for male patients vs. female patients by fracture location.

The average age at time of injury for female patients was 42 years, and the average age for male patients was 47 years. The average age at time of injury for female and male patients was further broken down (Fig. 13).

### Laterality

**Figure 14:**
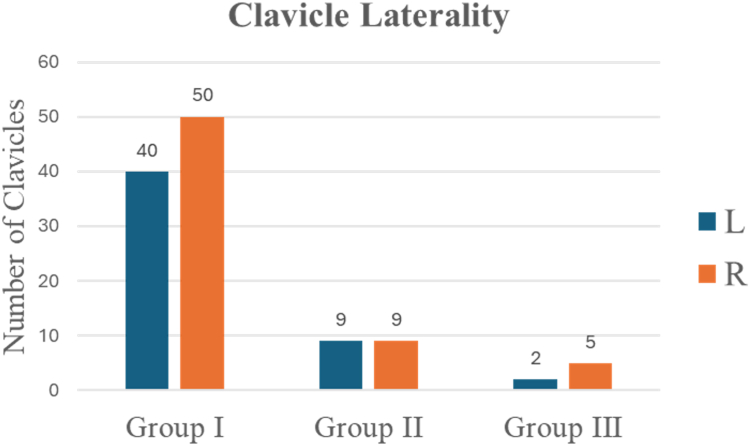
Clavicle laterality distribution by fracture group.

There were 51 left clavicle samples and 64 right clavicle samples (Fig. 14). There was no statistical significance difference between left and right clavicle shaft fracture location.

### Quantitative analysis

The goal of the image processing and numerical analysis was to mathematically define morphometric features of the clavicle and compare those values to where the fractures occurred. The initial sample size was 115 clavicles. One hundred clavicles from 100 patients were used for this portion of the analysis, and 15 were excluded due to severity of fractures that could not be properly skeletonized and mathematically analyzed. Of the 100 specimens included, there were 78 midshaft fracture specimens, 15 distal specimens, and 7 proximal fracture specimens. Several key variables were calculated are summarized in Tables II and III.

**Table II:** Measured variables amongst midshaft (group I), lateral (group II), and medial (group III) fractures.

Fracture location group	Medial radius of curvature in mm (STDV)	Medial arc length in mm (STDV)	Medial cord length in mm (STDV)	Medial angle in degrees (STDV)	Lateral radius of curvature (mm)	Lateral arc length in mm (STDV)	Lateral cord length in mm (STDV)	Lateral angle in degrees (STDV)
Group I (n = 78)	100.10 (41.96)	67.53 (17.27)	65.78 (17.00)	43.60 (15.22)	58.68 (31.68)	45.48 (12.93)	43.71 (12.94)	53.24 (21.69)
Group II (n = 15)	125.95 (54.80)	76.46 (20.97)	74.81 (20.18)	38.44 (13.78)	69.24 (31.26)	39.31 (11.41)	38.39 (11.06)	36.56 (16.86)
Group III (n = 7)	105.53 (33.31)	73.87 (17.58)	71.98 (17.07)	42.79 (13.73)	64.45 (34.62)	32.10 (14.01)	31.61 (13.82)	27.64 (18.68)
All groups (n = 100)	104.36 (44.12)	69.31 (18.02)	67.52 (17.67)	42.77 (14.85)	60.67 (31.72)	43.61 (13.24)	42.06 (13.08)	48.94 (22.47)

	Total arc length in mm (STDV)	Total cord length in mm (STDV)	Medial arc length to total arc length ratio (STDV)	Medial cord length to total cord length ratio (STDV)	Lateral arc length to total arc length ratio (STDV)	Lateral cord length to total cord length ratio (STDV)
Group I (n = 78)	113.00 (20.73)	109.42 (20.86)	0.59 (0.10)	0.60 (0.10)	0.41 (0.10)	0.40 (0.10)
Group II (n = 15)	115.76 (24.45)	113.21 (24.09)	0.66 (0.07)	0.66 (0.07)	0.34 (0.07)	0.34 (0.07)
Group III (n = 7)	105.97 (14.81)	103.58 (14.21)	0.70 (0.14)	0.69 (0.14)	0.30 (0.14)	0.31 (0.14)
All groups (n = 100)	112.92 (20.90)	109.58 (20.91)	0.61 (0.10)	0.61 (0.10)	0.39 (0.10)	0.39 (0.10)

*STDV*, standard deviation.

The variables measured were radius of curvature, arc length, cord length, angle, total arc length, total cord length, arc length to total arc length ratio, and cord length to total cord length ratio. All variables were measured for both the medial and lateral region of each clavicle.

**Table III:** Measured variables for female and male subjects.

Sex of subject	Medial radius of curvature in mm (STDV)	Medial arc length in mm (STDV)	Medial cord length in mm (STDV)	Medial angle in degrees (STDV)	Lateral radius of curvature (mm)	Lateral arc length in mm (STDV)	Lateral cord length in mm (STDV)	Lateral angle in degrees (STDV)
Female (n = 34)	97.67 (45.08)	60.33 (16.26)∗	58.97 (16.09)∗	45.94 (13.54)	57.25 (29.25)	43.74 (14.30)	42.35 (14.28)	44.24 (12.39)
Male (n = 66)	107.05 (43.36)	73.94 (17.22)∗	71.92 (16.91)∗	44.08 (14.62)	62.43 (33.00)	43.55 (12.77)	41.91 (12.52)	49.88 (19.53)

	Total arc length in mm (STDV)	Total cord length in mm (STDV)	Medial arc length to total arc length ratio (STDV)	Medial cord length to total cord length ratio (STDV)	Lateral arc length to total arc length ratio (STDV)	Lateral cord length to total cord length ratio (STDV)
Female (n = 34)	104.07 (18.39)∗	101.33 (18.57)∗	0.58 (0.12)∗	0.58 (0.12)∗	0.42 (0.12)∗	0.42 (0.12)∗
Male (n = 66)	117.49 (20.77)∗	113.84 (20.91)∗	0.63 (0.08)∗	0.63 (0.08)∗	0.37 (0.08)∗	0.37 (0.08)∗

*STDV*, standard deviation.

The variables measured were radius of curvature, arc length, cord length, angle, total arc length, total cord length, arc length to total arc length ratio, and cord length to total cord length ratio. All variables were measured for both the medial and lateral region of each clavicle.

∗ Indicates statistically significant values.

### Morphological analysis between groups

Total arc length and total chord length were calculated by taking the sum of the medical arc lengths and lateral arc lengths and the medical chord lengths and lateral chord lengths, respectively. Although the arc length is longer than chord length there is no statistically significant difference between the 2 measurements of length for all groups and between individual groups at *P* ≤.01. Going forward, we will be referencing the values of the arc lengths, keeping in mind the chord lengths are statistically similar. Other morphological values were calculated, including the radius of curvature (ROC), chord length, arc length, and angles for both the medial and lateral components of the clavicle.

The medial arc length to total arc length ratio represents how much of the clavicle length is defined by the medial portion. The same concept applies to the lateral arc length to total ratio. All values are references with respect to the medial (sternal) border of the clavicle as the starting point. The medial border was arbitrarily chosen. The inflection point of the clavicular curve is where the medial portion of the curve transitions to the lateral portion of the curve. Mathematically, it is where the fourth-degree polynomial curve switches from negative values to positive values. The medial portion of the clavicular curve compromises approximately 61% of the curve length, on average, with the lateral portion of the curve compromising the remaining 39% on average. The medial arc length to total arc length ratio was different between midshaft, lateral, and medial fracture groups; however, these values were not statistically significant between groups. Based on these findings, the inflection point can be approximated at 60% of the distance from the medial border of the clavicle.

### Morphological analysis between genders

Female patients, on average, had shorter total arc length and total chord length values compared to their male counterparts. The difference in clavicle length between female and male patients was found to be statistically significant and appears to be due to the difference in medial arc or cord length rather than the lateral arc and cord lengths, as only the medial arc and cord lengths had a statistically significant difference (Table III). Like the analysis conducted between fracture pattern groups, there was no significant difference found between the arc length values and the chord length values within the female or male subject data sets. There were no statistically significant differences between medial and lateral radii of curvature between male and female patients.

The differences between the female and male subject medial arc length to total length ratio were statistically significant at a *P* value < .05 (*P* = .02). The same was true for the lateral arc length to total arc length ratios between female and male patients as well (*P* = .02), meaning that where the inflection point occurs likely depends on the sex of the patient. There was no statistical significance at a *P* value < .05 between male and female patients regarding medial ROC, lateral ROC, lateral angle, and medial angle.

### Fracture distance analysis

The fracture distance was measured from the medial border of the clavicle for consistency. The total chord length was recorded and the ratio of the fracture distance to the total chord length was calculated (Tables IV and V).

**Table IV:** Fracture parameters by Allman Group were measured directly in the *Meshmixer* platform, using a calibrated measurement tool.

Fracture measurements by fracture location	Average total cord length in mm (STDV)	Average fracture distance from medial border in mm (STDV)	Average fracture distance to total cord length ratio
All groups (n = 115)	148.87 (13.99)	90.37 (27.44)	0.59 (0.17)
Group I (midshaft) n = 90	148.88 (14.47)	88.96 (12.54)	0.59 (0.07)
Group II (lateral or distal) n = 18	150.08 (12.53)	127.64 (16.11)	0.84 (0.11)
Group III (medial or proximal) n = 7	146.31 (11.87)	27.96 (11.08)	0.19 (0.07)

The fracture distance from the medial border was measured as the midpoint of the zone of comminution to the medial border of the clavicle.

**Table V:** Fracture parameters by female and male subjects were measured directly in the *Meshmixer* platform, using a calibrated measurement tool.

Fracture measurements by gender	Average total cord length in mm (STDV)	Average fracture distance from medial border in mm (STDV)	Average fracture distance to total length ratio
All groups (n = 115)	148.87 (13.99)	90.37 (27.44)	0.59 (0.17)
Female (n = 39)	139.72 (8.14)	82.16 (25.36)	0.59 (0.18)
Male (n = 76)	154.25 (13.65)	95.20 (27.70)	0.62 (0.15)

*STDV*, standard deviation.

The fracture distance from the medial border was measured as the midpoint of the zone of comminution to the medial border of the clavicle.

### Fracture length

There was a statistically significant difference in the mean fracture length between male and female subjects (Table VI).

**Table VI:** Average fracture length by subgroup.

Zone of communition by subgroup	Average total cord length in mm	Average zone of C (mm)	STDV (mm)	Zone of comminution as a percent of total clavicle length	Minimum fracture length (mm)	Maximum fracture length (mm)
Group I	148.88	30.61	18.16	20.56%	4.44	74.41
Group II	150.08	26.91	12.95	17.93%	9.61	54.45
Group III	146.31	24.47	12.13	16.72%	8.23	74.41
All groups	148.87	29.58	17.03	19.87%	4.44	74.41
Female	139.72∗	21.71∗	13.98	15.53%	4.44	70.10
Male	154.25∗	33.62∗	17.01	21.79%	4.44	74.41

*STDV*, standard deviation.

∗ Indicates statistically significant values.

## Discussion

Surgical management of clavicle fractures has increased in the past 2 decades as studies have shown improved functional outcomes.^7^^,^^14^^,^^16^ This shift has renewed interest in clavicle morphology, as understanding the anatomy facilitates surgical care. No previous study, to our knowledge, has attempted to define the clavicular inflection point and its relationship to high-energy fracture patterns.

Our findings support several correlations between fracture location and clavicle morphology. The medial arc and lateral arc connect to one another at the inflection point of the center curve. This inflection point occurs approximately 60% of the distance of the total clavicle length from the medial border. This finding was consistent with a parallel, ongoing study conducted by Orbay et al, in which the authors investigate clavicle morphology in dried human clavicle samples (J. Orbay, MD, unpublished data, December 2025). The authors mathematically defined the medial arc and lateral arc of the clavicular curve and found that the medial arc length is longer than the lateral arc length. Our data was consistent with this finding.

The medial ROC is larger than the lateral ROC, which corresponded to a smaller medial angle. With respect to angles, a smaller angle will correspond to a straighter curve, while a larger angle will correspond to a more dramatic curvature. Between male and female patients, there were no statistically significant differences between female ROC and medial angles compared to male ROC and medial angles, implying that the curvature of the clavicle may not depend on the sex of the patient. The length of the male clavicles was significantly longer than the female patient clavicles; however, only the medial arc length and medical cord lengths had statistically significant differences in length, whereas lateral arc and cord lengths did not. This alludes to the observation that the medial portion of the clavicle contributes to the length discrepancy between these 2 groups.

Previous studies of clavicle fractures have helped to discern where fractures occur based on gross morphology and ligamentous attachments. Ljunggren^9^ studied clavicle morphology with respect to functionality, noting that the junction between lateral and middle thirds is the thinnest point and is not reinforced by muscles or ligaments, making it more susceptible to injury. Crane et al^5^ determined whether the clavicle midshaft shows unique adaptation to atypical load-bearing when compared to the sternal (medial) and acromial (lateral) clavicular regions due to decreased Haversian remodeling in the middle third of the bone, especially with respect to the medial to lateral shape change. Both Ljunggren and Crane et al describe the gross anatomical region where the clavicular inflection point occurs but do not specify or define this point directly.

Few studies have investigated clavicle morphometric parameters in detail. Bachoura et al^3^ studied dried 3D skeleton clavicles samples and found that the length of the clavicle correlates with the midpoint cortical diameter and with the radius of medial curvature. Using healthy nonfractured clavicle specimens, Lambert et al^8^ also utilized CT based 3D modeling to discern morphometric relationships, finding that clavicular length, but not necessarily curvature, was the greatest variation in clavicle specimens. Agyeman et al^8^ looked closely at midshaft fractures in 166 patients post-operative radiographs, reporting 90% of midshaft fractures were centered lateral to the midpoint and 64% were completely lateral to the midpoint. The authors further defined a “fracture zone” localized 42% of the distance from the lateral border, or 58% percent from the medial border. Our results support this conclusion using 3D models derived from CT scans that were taken at the time of injury. The average fracture distance for midshaft fractures was located at a point corresponding to 60% of the total clavicular length when measured from the medial border. Our results continue to build from this concept by further correlating this location with the anatomical inflection point, an anatomical location not previously defined.

Clavicle morphology may affect clavicle shaft fracture fixation. Patel et al^10^ analyzed the efficacy of 2 popular precontoured superior anterior clavicle plates utilized in the fixation of displaced midshaft clavicle and determined poor fit in nearly 75% of cases. The authors' criteria for poor fit involved complete mismatch with at least one hole of the plate not over the bone, significant anterior or posterior overhang, and manual bending of the plate more than 30° to achieve fitting. A concrete geometric understanding of the medial and lateral arc ROC with relation to the clavicular inflection location may help to refine application of fixation principles. Furthermore, the average zone of comminution length is an important parameter of consideration that we analyzed in this study. Our findings demonstrate that midshaft clavicle fractures on average occur at length of 30.61 mm; however, there was a large range of values from a minimum of 4.44 mm and a maximum of 74.41 mm. Future directions of this work may include understanding morphological response to loading forces and determining specific trends in bone density, cortical thickness, and diameter at the clearly defined anatomical inflection point.

### Limitations

One of the limitations of this study is that it focuses on the midshaft region of the clavicle. Another limitation is the lack of information regarding hand dominance for all patients, which may have influenced the results, as the dominant extremity often experience different levels of stress or injury. The largest limitation of our study was that most of the patients were trauma patients with high-impact clavicle fractures requiring CT scans, which may not represent the broader spectrum of clavicle fractures, including those caused by lower-impact events. Selection bias may have been introduced due to the requirement for CT scans for inclusion, which inherently selected for patients with more severe or complex fractures typically seen in trauma cases, thus limiting the generalizability of the findings to less severe or nontraumatic clavicle fractures. Lastly, our methodology did not allow for analysis of all clavicle fractures in the cohort secondary to them not being able to be reconstructed, which introduced further selection bias.

## Conclusion

There was no statistically significant difference in fracture location between males and females (*P* = .74). The most common MOI was MVA, followed by motorcycle collision. The medial portion of the clavicular curve compromises approximately 61% of the curve length, on average, with the lateral portion of the curve compromising the remaining 39% on average. Based on these findings, the inflection point of the clavicle occurs at a length that is approximately 60% of the distance from the medial border of the clavicle. Using 3D models derived from CT scans that were taken at the time of injury, the average fracture distance for midshaft fractures was also located at a point corresponding to 60% of the total clavicular length when measured from the medial border. Our correlating this location with the anatomical inflection point, an anatomical location not previously defined. It can be inferred that midshaft fractures have a propensity to occur at the anatomical inflection point of the clavicle.

## Disclaimers

Funding: No funding was disclosed by the authors.

Conflicts of interest: The authors, their immediate families, and any research foundation with which they are affiliated have not received any financial payments or other benefits from any commercial entity related to the subject of this article.
